# How can regulation and reimbursement better accommodate flexible suites of digital health technologies?

**DOI:** 10.1038/s41746-024-01156-y

**Published:** 2024-07-02

**Authors:** Rebecca Mathias, Peter McCulloch, Anastasia Chalkidou, Stephen Gilbert

**Affiliations:** 1 Else Kröner Fresenius Center for Digital Health, TUD Dresden University of Technology, Dresden, Germany; 2 Nuffield Department of Surgical Sciences, University of Oxford, Oxford, UK; 3 National Institute for Health and Care Excellence (NICE), London, UK

**Keywords:** Health policy, Health care economics

## Abstract

Individual digital health devices are increasingly being bundled together as interacting, multicomponent suites, to deliver clinical services (e.g., teleconsultation and ‘hospital-at-home services’). In the first article of this two-article series we described the challenges in implementation and the current limitations in frameworks for the regulation, health technology assessment, and reimbursement of these device suites and linked novel care pathways. A flexible and fit-for-purpose evaluation framework that can analyze the strengths and weaknesses of digital technology suites is needed. In this second article we describe adaptations that could enable this new technological paradigm while maintaining patient safety and fair value.

The delivery of healthcare is evolving to incorporate digital health technology (DHT) with a rise in interacting systems of DHT suites, characterized by modularity, embedding, aggregation, adaptability, co-design, and rapid evolution. This ongoing transformation has led to a shift from conventional face-to-face patient-clinician interactions to telemedicine approaches that incorporate aspects of remote health care provider (HCP) management of patients, the greater use of wireless sensor technologies for measuring patient physiology, and the merging of these concepts through the inclusion of aspects of automation, machine decision making and even advice^1^. This entails the exchange of patient data, data and algorithm-driven practices, and distributed trust extending beyond traditional healthcare practices^2^. Our previous article^3^ in this two-article series, described how there is much enthusiasm for the potential of these suite-based workflows to positively transform care, alongside substantial challenges in their regulation, health technology assessment (HTA), and reimbursement. Frameworks are unsurprisingly playing catch up with these fast-evolving technologies. Some device suites are specifically developed for aggregated use. Increasingly, individually approved “standalone” DHTs, which may even have “standalone” HTA and reimbursement recommendations, can be brought together on an ad hoc basis. Understandably, current evaluation frameworks focus on individual DHTs rather than on integrated systems or bundles. The US Food and Drug Administration (FDA) commissioner Robert Califf directly acknowledged this in a February 2024 speech, saying “If you think about device development, like sensors, if you do it one at a time, you’ll never put a whole suite together into something that fits together in the [home] environment. [The FDA is] working on a strategy on how to create regulatory pathways to help make that happen”^4^.

A range of different types of DHT aggregations are possible for which differing regulatory and HTA considerations apply. Some of these suites are well established, and some exist more as concepts described in the literature rather than as approaches currently adopted in mainstream healthcare (Table 1). We describe the need for change in current frameworks, and describe approaches that could be applied for this relatively new phenomenon.

**Table 1:** Examples of aggregated suites of DHTs in healthcare

#	Description of functional aggregate of DHTs	Example system or service	Usual regulatory and HTA status of system
1	Single manufacturer developed system of DHTs, as interacting components of a system, each with individual MD approval or MD approval as a single system, together designed to interact to deliver a specific function.	An implanted pacemaker (modern forms of which combines DHT interactivity with an older closed-loop stimulator concept) has interaction with: (i) remote monitoring device; (ii) patient app; (iii) remote monitoring HCP platform; (iv) HCP pacemaker programmer.	Regulatory- MD HTA/reimbursement- Generally, as a system.
2	Multi-manufacturer developed DHTs, each approved as MDs. Brought together and placed together on the market as a system by a single commercial entity and supplied to multiple health systems.	A series of DHTs brought together to create a remote smart clinic platform and designed to enable remote physical exams by clinicians in the patient’s home. DHTs would typically include thermometers, stethoscopes, otoscopes, laryngoscope, all integrated through a mobile phone or tablet-based platform^11^.	Regulatory- MD HTA/reimbursement - Generally, as a system.
3	Multi-manufacturer developed DHTs grouped together with a combination of other MD types, non-MDs, medical IT systems (e.g., EHR), and human digital interactions - e.g., teleconsultation system and doctors. Brought together and placed together on the market as a system by a single commercial entity and supplied to multiple health systems.	A HaH system, including a patient facing app, a clinical dashboard for monitoring patient vitals and alert systems to flag abnormalities, developed by a commercial operator. Platforms include smartphone apps with wearable kits and monitoring devices that are tailored to the patient’s care pathway^12,13^.	Regulatory- Includes MD, non-MD DHT, and human components. HTA/reimbursement- Generally, as a system.
4	Multi-manufacturer developed DHTs grouped together with a combination of other MD types, non-MDs, medical IT systems (e.g., EHR), and human digital interactions - e.g., teleconsultation system and doctors. Brought together automatically, flexibly and dynamically, making use of existing consumer wearables in smartphones, smartwatches and smartphones.	Bring Your Own Device or BYOD is a concept gaining some traction where patients can use their own devices like smart phones linked with a medical device for point of care diagnostics or communication of results with their HCP^14^.	Regulatory- Includes MD, non-MD DHT and human components. HTA/reimbursement: - Not yet known.

The suites are described, along with examples of the medical settings, applications, and workflows in which they are used. The regulatory status of example DHT suites are summarized as is their most common treatment in HTA and reimbursement. There is significant overlap between categories, with the main difference currently being the regulatory techniques adopted by manufacturers, often due to ambiguity rather than technical variations between products.

*MD* Medical device, *HCP* Health care provider, *HAH* Hospital at Home, *EHR* Electronic Health Record.

## Regulatory considerations for aggregates of DHTs

It is rational that medical device regulation frameworks require developers to consider the interactions between their devices and other devices that they are planned to directly interact with^5^. In former eras, it is likely that the developer could anticipate all future uses and DHT interactions. Although this approach may have seemed logical and balanced at the time of writing the laws, the increasing networked interaction of DHTs now falls short in addressing the complexities of modern mobile communications systems. While we recognize that communication systems originated in low-risk environments compared to healthcare, to keep pace with advancing digital progress a cautious introduction of systems with safeguards to identify unintended consequences early on, could be necessary. This approach prioritizes dynamic monitoring and evidence generation over aversion to technology, promoting optimal and safe healthcare practices without advocating for a free-for-all system.

As highlighted in Table 1, it is highly challenging to define a taxonomy for suites of devices. There is need for understanding both from the manufacturer’s and regulator’s side to strike a balance that is proportionate to risk. In this complex environment, transparency is key and requires to be noted by both parties. Manufacturers have the responsibility to make data available and conduct trend reporting for real-world performance monitoring to ensure patient safety. The pandemic recognized the need for aggregation of individual DHTs into service bundles but without systematic and automated monitoring that can now be achieved. Flexible monitoring ideas, like integrated collection of feedback from system users (patients and HCPs), can support this effort^1,6^.

## The need for a flexible way forward in HTA evaluation of DHT aggregates

Just as regulation needs to adopt a more flexible approach to governing interacting DHT systems, the same adaptability is necessary for HTA and reimbursement processes. There is currently no comprehensive assessment framework to evaluate DHTs in general, let alone modular DHT suites that function independently or in an aggregated system. Current DHT assessment approaches, which are based on care processes, could evolve to be system-based. For example, in Belgium, authorized digital health applications may receive reimbursement as part of a healthcare process, receiving a lump sum for treatment rather than individual reimbursements for specific actions^7^. This model can be applied to modular digital health tools, reimbursing them as part of a whole system instead of as individual components.

Another route to promote innovation in healthcare technology was adopted by Germany proposing the digital health application (DiGA) fast-track system that allowed temporary reimbursement, during a one-year evidence generation period, and this did achieve its aim^8^. There were also drawbacks—full reimbursement was provided to many DiGAs that later did not prove positive benefits^9^, but soon to be enforced rules on ongoing performance monitoring, bring promise of a fair balance of early reimbursement and promotion of innovation^10^. Our view is that the described model could be extended to the provisional approval of integrated DHT suites, allowing temporary approvals and partial reimbursement, while sharing risks with manufacturers until benefits are proven. To deliver safety, it would be essential to ensure that: (i) manufactures define an ‘envelope’ for the safe use of their device—which may not predict every device combination but delineates permissible use types, such as suitability for critical monitoring; and, (ii) the registration of new combinations and use cases as they are identified; and, (iii) the testing of new use cases and combinations promptly, automatically gathering feedback on use where possible^1^ and with the requirement for manufactures to risk assess and gather surveillance data.

## Summary

We advocate for better recognition in regulations and HTA frameworks that DHTs can be aggregated into modular suites, expanding beyond discrete purposes. Here the individual device intended purposes may form a guide but should not be a barrier to use in wider, combinatory use cases. Why is this needed? Nations will get the digital care futures they legislate for, regulate for and choose through reimbursement decisions. To enable transformational benefit through new care paradigms that are possible through digital medicine, a first step is the acknowledgment and readiness for change. Under-cautious approaches could lead to unsafe technologies. Regulatory, HTA and reimbursement approaches based on traditional concepts of discrete DHTs, are unlikely to realize the comprehensive benefits of digital transformation. Countries adopting flexible approval, assessment, and payment frameworks, coupled with robust and intelligent oversight, are likely to achieve digitally transformed medical technologies suited to societal needs, reducing costs, environmental impact, and enhancing quality of care.
